# Post-sphincterotomy bleeding: fully-covered metal stents for hemostasis

**DOI:** 10.12688/f1000research.2-171.v1

**Published:** 2013-08-12

**Authors:** Anthony T DeBenedet, Grace H Elta

**Affiliations:** 1 Division of Gastroenterology and Hepatology, Department of Internal Medicine, University of Michigan Medical Center, Ann Arbor, MI, 48109, USA

## Abstract

**Background/objectives**: In endoscopic retrograde cholangiopancreatography, post-sphincterotomy bleeding (PSB) is a common complication of biliary sphincterotomy. Recently, the temporary placement of fully-covered metal stents (FCMS) into the biliary tree in order to achieve a tamponade effect has been described as an additional therapeutic option for PSB. The aim of this article is to review the literature on FCMS for hemostasis in PSB and update the treatment algorithm for this complication.

**Methods**: A PubMed literature search was conducted using the search terms post-sphincterotomy, bleeding, and stent. 33 articles were reviewed, along with their references, and four were found to describe the use of FCMS for hemostasis in PSB.

**Results**: A total of 21 patient cases were described in the four articles. All patients received FCMS for PSB hemostasis following the application and subsequent failure of traditional therapies (conventional pharmacologic injection, thermal or electrocoagulation, and mechanical therapy (balloon tamponade or endoclip)). Successful hemostasis was achieved in all patients through FCMS placement. No major complications were observed.

**Conclusion**: These 21 cases demonstrate that FCMS are a viable therapeutic option for PSB.  It is reasonable to consider stent placement for patients in which traditional interventions fail in order to avoid the need for angiographic or surgical hemostasis.

## Introduction

In endoscopic retrograde cholangiopancreatography (ERCP), post-sphincterotomy bleeding (PSB) is a common complication of biliary sphincterotomy. The incidence varies from 1–48% depending on what definition is applied, specifically whether bleeding *during* the procedure is included ^1^; intraprocedural bleeding is classified as immediate, whereas bleeding that occurs after the procedure is considered delayed ^2– 4^. The accepted criteria for grading PSB are: mild, which is a hemoglobin drop of less than 3 grams and no transfusion requirement; moderate, which is a transfusion requirement of 4 units or less and no angiographic intervention or surgery; and severe, which is a transfusion requirement of 5 units or more or intervention (angiographic or surgical) ^2^.

Immediate bleeding will often spontaneously stop without endoscopic intervention. But for immediate or delayed PSB that requires intervention, the endoscopic hemostasis options parallel those for bleeding elsewhere in the gastrointestinal tract: conventional pharmacologic injection (epinephrine); thermal or electrocoagulation; and mechanical therapy (balloon tamponade or endoclip). With the exception of pharmacologic injection, it can be challenging to effectively administer these various interventions through a side-viewing endoscope; however, endoscopic hemostasis is usually successful. When hemostasis is not achieved, angiographic embolization or surgery is required.

Recently, the temporary placement of fully-covered metal stents (FCMS) into the biliary tree to achieve a tamponade effect has been described as an additional therapeutic option for PSB ^5– 8^. The aim of this article is to review the literature on FCMS for hemostasis in PSB and update the treatment algorithm for this complication ^1^.

## Literature review

A PubMed literature search was conducted in July 2013 using the search terms post-sphincterotomy, bleeding, and stent. 33 articles were reviewed, along with their references, and four were found to describe the use of FCMS for hemostasis in PSB.

In 2010, Pisa and colleagues were the first to describe the technique in a two-patient case series that included one adult and one pediatric patient ^5^. The adult initially received epinephrine injection and endoclip placement, but bleeding persisted. A plastic stent (10 Fr, 5 cm, OASIS, Wilson Cook Medical, Winston-Salem USA) was then placed, which also did not achieve hemostasis. Thus, a fully-covered metallic stent (4 cm in length and 1 cm in diameter, Wallstent, Boston Scientific, Natik USA) was placed. No further bleeding occurred. The stent was removed four weeks later without complication. The child received the same metal stent, after initially failing epinephrine injection and balloon tamponade. This stent was removed three weeks later without complication.

Shah and colleagues were next to describe the use of FCMS for hemostasis in PSB ^6^. Their case series included five adult patients who failed various traditional therapies prior to stent placement (epinephrine injection, thermocoagulation, endoclip, and angiographic embolization). Each patient received a stent that was sized according to the patient’s anatomy (4–6 cm in length and 8–10 mm in diameter, Wallstent, Boston Scientific, Natik USA). The stents were placed so that the proximal end did not obstruct the cystic duct orifice and the distal end extended 1–2 cm beyond the biliary orifice. For each patient, stent removal was scheduled 2–4 weeks following stent placement. In three of the five patients, the stents were removed without complications; in two patients, the stents were not seen at the time of removal and were presumed to have migrated distally, as there was no fluoroscopic evidence of stent retention.

Itoi *et al.* published their case series of 11 patients in 2011 ^7^. All patients had bile duct stones and had failed either mono- or combined therapy (hypertonic saline epinephrine injection, balloon tamponade, and/or endoclip placement) prior to stent placement. The FCMS were 6 cm in length and 10 mm in diameter (Covered Wallstent or Covered WallFlex, Boston Scientific Tokyo, Japan or Combistent, Taewoong, Seoul South Korea). Complete hemostasis was achieved in all patients who received stent placement, and stents were left in place for approximately one week. All were removed successfully without complication (one stent had migrated to the duodenum).

Finally, Valats and colleagues described three patients with PSB who received various sized FCMS (Biliary Wallflex; Boston Scientific, Natick USA), which resulted in successful hemostasis ^8^. These patients had also failed conventional endoscopic treatment prior to stent placement. No complications were observed. This case series also described several cases of common bile duct trauma causing hemobilia that were subsequently treated with FCMS.

## Discussion

These 21 cases demonstrate that FCMS are a viable therapeutic option for PSB. However, there are several important issues that remain unanswered, including: which patients should be considered for stents, optimal stent size, timing of stent removal, the frequency of complications from stent placement, and whether stent placement is cost-effective.

It is reasonable to consider stent placement in patients who fail traditional interventions to avoid surgery or angiographic hemostasis. Whether stents should be used earlier in the hemostasis algorithm is unclear. It has been suggested that stenting could be considered prior to traditional interventions in patients with severe bleeding, a history of coagulopathy, and/or in patients who have required multiple endoscopic procedures for hemostasis ^9^. This approach may also be both clinically and financially more effective.

With regard to stent size, the diameter should be large enough to avoid upstream migration into the bile duct or downstream displacement into the duodenum. Stent migration occurred in three of the 21 cases reviewed for this article, which is congruent with migration rates published for other conditions ^10– 13^. However, migration rates may actually be higher in the setting of PSB compared to biliary strictures because of the biliary sphincterotomy, which theoretically fosters a looser "fit" within the biliary tree. Itoi *et al.* suggested 10 mm as the ideal diameter ^7^. Smaller diameter stents may become dislodged and may also not effectively achieve tamponade. The distal end of the stent should be beyond the biliary orifice to ensure effective tamponade, and the proximal end, if possible, should be distal to the cystic duct takeoff to theoretically reduce the risk of cholecystitis, although this risk is uncertain and may even be non-existent ^14, 15^.

When placing covered stents in general, there is also a risk of pancreatic duct outflow obstruction which may potentially cause pancreatitis. This has been reported in up to 7% of patients in a previous study ^12^. There were no reported cases of pancreatitis when placing stents for PSB in the cases reviewed for this article. It is possible that having a biliary sphincterotomy decreases the risk of pancreatitis in the setting of stent placement because there is less pressure from the stent on the pancreatic orifice ^16^. Shah *et al*. suggest that stents should be removed within 2–4 weeks of placement ^6^.

From a cost perspective, there are no published cost-effectiveness analyses on FCMS for PSB. There are certainly costs associated with the placement and removal of FCMS. However, if stent placement prevents the need for angiographic embolization or surgery then there would hypothetically be cost-savings. Similarly, if stent placement is pursued as a first-step in patients who are high-risk for recurrent bleeding or complications, this could also be cost-saving ^7^.

In conclusion, FCMS are a viable therapeutic option for the treatment of PSB that is refractory to traditional endoscopic interventions. The optimal patient population for this intervention, as well as the optimal stent size and removal time should be determined on a case-by-case basis until more data becomes available.

## References

[1] Ferreira LE Baron TH: Post-Sphincterotomy Bleeding: Who, What, When, and How. *Am J Gastroenterol.* 2007;102(12):2850–2858 10.1111/j.1572-0241.2007.01563.x 18042116

[2] Cotton PB Lehman G Vennes J: Endoscopic sphincterotomy complications and their management: An attempt at consensus. *Gastrointest Endosc.* 1991;37(3):383–93 10.1016/S0016-5107(91)70740-2 2070995

[3] Freeman ML Nelson DB Sherman S: Complications of endoscopic biliary sphincterotomy. *N Engl J Med.* 1996;335(13):909–18 10.1056/NEJM199609263351301 8782497

[4] Freeman ML: Understanding risk factors and avoiding complications with endoscopic retrograde cholangiopancreatography. *Curr Gastroenterol Rep.* 2003;5(2):145–53 10.1007/s11894-003-0084-9 12631456

[5] Di Pisa M Tarantino I Barresi L: Placement of covered self-expandable metal biliary stent for the treatment of severe postsphincterotomy bleeding: outcomes of two cases. *Gastroenterol Res Pract.* 2010 10.1155/2010/138748 20631831 PMC2901612

[6] Shah JN Marson F Binmoeller KF: Temporary self-expandable metal stent placement for treatment of post-sphincterotomy bleeding. *Gastrointest Endosc.* 2010;72(6):1274–78 10.1016/j.gie.2010.08.012 20951987

[7] Itoi T Yasuda I Doi S: Endoscopic hemostasis using covered metallic stent placement for uncontrolled post-endoscopic sphincterotomy bleeding. *Endoscopy.* 2011;43(4):369–372 10.1055/s-0030-1256126 21360425

[8] Valats J Funakoshi N Bauret P: Covered self-expandable biliary stents for the treatment of bleeding after ERCP. *Gastrointest Endosc.* 2013;78(1):183–187 10.1016/j.gie.2013.02.035 23587846

[9] Ferreira LE Fatima J Baron TH: Clinically significant delayed postsphincterotomybleeding: a twelve year single center experience. *Mineva Gastroenterol Dietol.* 2007;53(3):215–223 17912183

[10] Traina M Tarantino I Barresi L: Efficacy and safety of fully covered self-expandable metallic stents in biliary complications after liver transplantation: a preliminary study. *Liver Transpl.* 2009;15(11):1493–8 10.1002/lt.21886 19877248

[11] Cahen DL Rauws EA Gouma DJ: Removable fully covered self-expandable metal stents in the treatment of common bile duct stricture due to chronic pancreatitis: a case series. *Endoscopy.* 2008;40(8):697–700 10.1055/s-2008-1077353 18704837

[12] Mahajan A Ho H Sauer B: Temporary placement of fully covered self-expandable metal stents in benign biliary stricture: midterm evaluation (with video). *Gastrointest Endosc.* 2009;70(2):303–9 10.1016/j.gie.2008.11.029 19523620

[13] Kahaleh M Behm B Clarke BW: Temporary placement of covered self-expandable metal stents in benign biliary strictures: a new paradigm (with video)? *Gastrointest Endosc.* 2008;67(3):446–54 10.1016/j.gie.2007.06.057 18294506

[14] Fumex F Coumaros D Napoleon B: Similar performance but higher cholecystitis rate with covered biliary stents: results from a prospective multicenter evaluation. *Endoscopy.* 2006;38(8):787–92 10.1055/s-2006-944515 17001568

[15] Telford JJ Carr-Locke DL Baron TH: A randomized trial comparing uncovered and partially covered self-expandable metal stents in the palliation of distal malignant biliary obstruction. *Gastrointest Endosc.* 2010;72(5):907–14 10.1016/j.gie.2010.08.021 21034891

[16] Simmons DT Petersen BT Gostout CJ: Risk of pancreatitis following endoscopically placed large-bore plastic biliary stents with and without biliary sphincterotomy for management of postoperative bile leaks. *Surg Endosc.* 2008;22(6):1459–63 10.1007/s00464-007-9643-8 18027045

