# Supplementary material for: Post-sphincterotomy bleeding: fully-covered metal stents for hemostasis
Source: F1000Res. 2013 Aug 12;2:171. [Version 1] doi: 10.12688/f1000research.2-171.v1 (PMC3869479; doi:10.12688/f1000research.2-171.v1)
Supplement: Peer review history

# Peer review: Referee response for version 1

**Published:** 2013-11-11

**Authors:** David Carr-Locke

**Affiliations:** 1 Division of Digestive Diseases, Beth Israel Medical Center, New York, NY, USA

Severe post-sphincterotomy bleeding during or after ERCP requiring intervention other than simple measures, such injection of 1:10,000 epinephrine, or that does not respond to first line attempts at hemostasis, is uncommon. This review, by DeBenedet and Elta, describes 21 patients from four articles where fully-covered self-expanding metallic stents were placed to provide tamponade to achieve successful hemostasis in 100% without complication. All stents were subsequently removed endoscopially or had passed spontaneously. This is a highly select group of patients but this option is a useful addition to the range of hemostatic modalities available for this difficult situation.

I have read this submission. I believe that I have an appropriate level of expertise to confirm that it is of an acceptable scientific standard.

# Peer review: Referee response for version 1

**Published:** 2013-09-19

**Authors:** Guido Costamagna

**Affiliations:** 1 Catholic University of Rome, Rome, Italy

The article provides a precise review of a "salvage" technique for refractory post-sphincterotomy bleeding, the discussion underlines the pro and cons of the technique, and the article can be useful for the endoscopic community.

I have read this submission. I believe that I have an appropriate level of expertise to confirm that it is of an acceptable scientific standard.
